# Is conduction system pacing a panacea for pacemaker therapy?

**DOI:** 10.1080/17434440.2024.2370827

**Published:** 2024-06-24

**Authors:** Stephe Kamalathasan, Maria Paton, John Gierula, Sam Straw, Klaus K. Witte

**Affiliations:** a Cardiology Department, Leeds Teaching Hospitals NHS Trust, Leeds, UK; b Leeds Institute of Cardiometabolic Medicine, University of Leeds, Leeds, UK

**Keywords:** Conduction system pacing, heart failure, cardiac resynchronization, therapy, His-bundle pacing, left bundle branch area pacing, bradycardia

## Abstract

**Introduction:**

While supported by robust evidence and decades of clinical experience, right ventricular apical pacing for bradycardia is associated with a risk of progressive left ventricular dysfunction. Cardiac resynchronization therapy for heart failure with reduced ejection fraction can result in limited electrical resynchronization due to anatomical constraints and epicardial stimulation. In both settings, directly stimulating the conduction system below the atrio-ventricular node (either the bundle of His or the left bundle branch area) has potential to overcome these limitations. Conduction system pacing has met with considerable enthusiasm in view of the more physiological electrical conduction pattern, is rapidly becoming the preferred option of pacing for bradycardia, and is gaining momentum as an alternative to conventional biventricular pacing.

**Areas covered:**

This article provides a review of the current efficacy and safety data for both people requiring treatment for bradycardia and the management of heart failure with conduction delay and discusses the possible future roles for conduction system pacing in routine clinical practice.

**Expert opinion:**

Conduction system pacing might be the holy grail of pacemaker therapy without the disadvantages of current approaches. However, hypothesis and enthusiasm are no match for robust data, demonstrating at least equivalent efficacy and safety to standard approaches.

## 1. Introduction

Heart failure, conduction delay, and pacing are closely related. On the one hand, the most concerning complication of long-term pacing for bradycardia is heart failure, while, on the other hand, conduction delay (especially in the form of left bundle branch block) in those with existing heart failure is associated with less response to medical therapy and worse outcomes [1]. Conduction system pacing (CSP) is an alternative strategy to right ventricular pacing in which ventricular stimulation is delivered to the intrinsic conduction system and is rapidly becoming the preferred option for right ventricular lead position [2]. CSP includes both His-bundle pacing, in which electrical stimulation occurs at the bundle of His and left bundle branch area pacing (LBBAP) in which stimulation occurs more distally, in the area of the left bundle at ventricular level. It is proposed that these approaches avoid the dyssynchrony characteristic of stimulation from apical lead positions and might therefore reduce the risks of pacing-associated left ventricular dysfunction, and heart failure. CSP has also been proposed as an intriguing alternative to coronary sinus lead placement in patients with heart failure and intrinsic conduction delay, for whom biventricular pacing (cardiac resynchronization therapy; CRT) is currently indicated; both for those in whom the implantation of a coronary sinus lead is not possible and as a more physiological alternative to conventional biventricular pacing [3]. The present review will summarize the current evidence and outline the advantages and possible disadvantages of the more widespread adoption of conduction system pacing for these indications.

## 2. Heart failure

### 2.1. The burden of heart failure

Heart failure with a reduced left ventricular ejection fraction (HFrEF) is a common and incurable condition, characterized by symptoms of breathlessness and exercise intolerance. The prevalence of HFrEF is increasing in developed countries, with much of this related to aging populations [4]. In addition to contributing to adverse effects on the individual, heart failure has a significant societal burden and costs for both inpatient and community-based care [5]. Hence, optimal medical and device therapies for HFrEF, and perhaps more importantly, its prevention, are critical. The last two decades have seen dramatic improvements in outcomes for those living with HFrEF, in part due to the development and implementation of device-based therapies including CRT and implantable cardioverter defibrillators (ICD) [6].

### 2.2. Why does pacing for bradycardia cause heart failure?

Pacemaker implantation for bradycardia improves quality of life [7] and survival [8], with over a million devices implanted globally each year [5]. The most common approach to ventricular lead placement remains the right ventricular apex. Implantation in this position is safe and well tolerated, with currently available leads having shown longevities extending beyond 20 years. However, electrical stimulation of the right ventricular apex results in dyssynchronous left ventricular activation and, in the longer-term, adverse remodeling. Hence, long-term right ventricular apical pacing results in an adverse effect on left ventricular function [9,10] can lead to the development of the heart failure syndrome [11], and as a consequence, a worse prognosis [12,13].

Although the burden of right ventricular pacing appears to be a key driver of progressive left ventricular remodeling, the effect is modified by the presence of other co-morbidities. Those who have significant cardiac disease are more likely to have conduction tissue disease [3], while the majority of people exposed to a high right ventricular pacing burden will never go on to develop overt left ventricular dysfunction or heart failure. Hence, progressive remodeling is not exclusively the fault of the lead position, such that the term pacing-*associated* heat failure is becoming more widely accepted than pacing-*induced* heart failure. Nevertheless, the adverse relationship between right ventricular pacing and left ventricular dysfunction is supported by evidence that the reduction of paced beats by careful device reprogramming, including the use of right ventricular pacing avoidance algorithms, improves left ventricular function without compromising quality of life [14–17].

### 2.3. What strategies exist to treat pacing-associated heart failure?

For many patients with high degree atrio-ventricular block, avoiding a high ventricular pacing burden is not possible even with modern pacing avoidance algorithms. For these individuals, there are now robust data showing that optimal medical therapy for heart failure and, where necessary, an upgrade to biventricular pacing are associated with improved clinical outcomes [18,19]. However, the holy grail for pacing for bradycardia remains the ability to deliver *de novo* heart rate support in a form which eliminates these risks and avoids the need for medical therapy or further procedures. The difficulties surrounding predicting which patients will go on to experience the deleterious effects of right ventricular pacing make any personalized approach challenging. Hence, one option is to offer some form of more physiological ventricular activation to all. Such a pacing strategy would need to be sufficiently reliable and safe that it could be offered routinely to an unselected population.

### 2.4. What strategies already exist to prevent pacing-associated heart failure?

The last 20 years have seen the rise and fall of right ventricular septal pacing and preemptive biventricular pacing as attempts to mitigate the risks of left ventricular remodeling in those requiring pacemaker implantation for bradycardia. Neither has consistently demonstrated significant benefits in patient-orientated outcomes in populations thought to be at risk of adverse events. The Protect-Pace study randomized 240 patients with high-degree atrioventricular block and normal left ventricular ejection fraction (LVEF) requiring >90% right ventricular pacing to an apical or high septal lead position [20]. Both techniques were associated with modest reductions in LVEF (~2%) with no between-group differences at 2-years and only minor differences in global longitudinal strain between the two groups [21]. This study and others have demonstrated increased procedural and fluoroscopy times for septal lead placement [22] and that the risk of atrial fibrillation thought to be associated with right ventricular apical pacing, was in fact related to pre-implant changes in left atrial structure and function, and not right ventricular lead location [23]. Therefore, the only robust clinical benefit of septal lead placement might be the reduced risk of perforation, a rare but potentially fatal complication of the standard apical approach [24].

Biventricular pacing restores left ventricular synchrony in those with left bundle branch block, which led to the plausible concept that *de novo* implantation of these devices for patients likely to require a high burden of right ventricular pacing for bradycardia could prevent progressive adverse left ventricular remodeling. Initial pilot studies using a cross-over design were promising, with both HOBIPACE (Homberg Biventricular Pacing Evaluation) and COMBAT (Conventional versus Multisite Pacing for BradyArrhythmia Therapy) demonstrating that in those receiving pacemaker implantation who also had LVEF ≤ 40%, the receipt of CRT was associated with better left ventricular function and exercise tolerance compared to right ventricular pacing [25,26]. The lack of adverse remodeling with ‘up front’ biventricular pacing is a consistent finding across smaller studies [27,28], with some even demonstrating improved symptoms, exercise capacity [29], and clinical outcomes [30], although the effect on long-term progressive remodeling and adverse outcomes is less clear.

Subsequent evaluation in two large clinical trials reached conflicting results. BLOCK-HF (Biventricular versus Right Ventricular Pacing in Heart Failure Patients with Atrioventricular Block) included 918 people with an indication for pacing and LVEF ≤ 50% to receive a device capable of biventricular pacing (with or without a defibrillator) and randomized participants to biventricular or right ventricular pacing [31]. The primary combined endpoint of all-cause mortality, requirement for intravenous diuretic therapy or a ≥15% increase in left ventricular end-systolic volume index were observed less frequently in those randomized to biventricular pacing (hazard ratio 0.74 [0.60–0.90]). The individual components of the primary outcome were also supportive of a strategy to consider *de novo* cardiac resynchronization therapy for these patients. However, the study design in which all patients received a cardiac resynchronization therapy device prior to randomization mean that it was impossible to assess the net benefit, given that coronary sinus lead implantation is associated with its own unique complications and increased procedure times [32]. The case for unselected ‘up front’ cardiac resynchronization therapy was examined in BIOPACE (Biventricular pacing for Atrioventricular Block to Prevent Cardiac Desynchronization) which randomized 1810 patients regardless of left ventricular function (1239 had LVEF > 50%) and significant atrioventricular block (PR interval >220 ms, type II or III atrioventricular block) to receive cardiac resynchronization therapy or standard right ventricular pacing. BIOPACE was neutral for the combined endpoint of death or heart failure hospitalization despite mean follow-up of 5.6 years, regardless of baseline LVEF [33]. Given the conflicting evidence of prognostic benefit, current guidelines provide a somewhat pragmatic class I indication for the ‘up front’ implantation of cardiac resynchronization therapy for patients who require ventricular pacing for bradycardia and have LVEF < 40%, regardless of symptoms, while His-bundle pacing is given a cautious IIb recommendation in those with LVEF ≥ 40% likely to require >20% right ventricular pacing [34].

Despite the well-recognized deterioration in left ventricular function with right ventricular pacing, most patients without existing left ventricular dysfunction who receive standard pacemaker therapy will never develop clinical heart failure. This is true even for those who require near 100% right ventricular pacing, which itself is poorly predictable even when the baseline indication is third degree atrioventricular block [35]. For those that do develop some degree of impaired left ventricular function as a result of right ventricular pacing, the risks of heart failure hospitalization are at worst modestly elevated and can be reduced by the provision of optimal medical therapy [18]. Hence, any treatment which tries to avoid this complication needs to be safe, straightforward, and reliable since a small increase in complication rates resulting from a more complex, time-consuming procedure is likely to negate any benefits in unselected populations.

## 3. Bradycardia

### 3.1. His-bundle pacing for bradycardia

First described more than two decades ago, His-bundle pacing is an approach in which the His-Purkinje conduction system is directly stimulated by an active fixation lead implanted into the intraventricular septum at the bundle of His, using fluoroscopy and a His electrocardiogram to guide position [36]. In the initial case series describing the technique, successful His-bundle pacing was achieved in only 67% of patients, limited by a lack of purpose-built catheters as well as high acute and chronic pacing thresholds. More recent studies have demonstrated higher implant success rates, albeit less often achieved in those with existing interventricular conduction delay [37]. Where successful, this technique achieves rapid, physiological, and coordinated ventricular depolarization, with the promise of avoiding the adverse left ventricular remodeling associated with apical lead positions. In a small randomized cross-over trial enrolling 38 patients with atrioventricular block, a narrow intrinsic QRS and ‘preserved’ (>40%) LVEF, His-bundle pacing resulted in better left ventricular function (LVEF 55 ± 10% vs 50 ± 11%; *p* = 0.005) and measures of mechanical synchrony, although with no differences in any patient-orientated outcomes, and at the expense of significantly higher pacing thresholds [38].

These advantages might make His-bundle pacing particularly attractive for patients without intrinsic conduction delay undergoing atrio-ventricular node ablation for atrial fibrillation [39]. However, despite advances in pacing technology, high acute and chronic pacing thresholds remain a consideration, and together with the risk of lead displacement [40–43] mean His-bundle pacing is not recommended by current guidelines without an additional right ventricular ‘back up’ lead [34]. Larger studies are still required to confirm whether these benefits translate into clinical outcomes, and to define the indications for which it might be advantageous. Additional technical challenges include the proximity of the septal leaflet to the tricuspid valve, the membranous interventricular septum making siting of the lead challenging, and the fact that many patients have conduction disease distal to the bundle of His.

### 3.2. Left bundle branch area pacing for bradycardia

The first case of direct left bundle branch pacing was reported in 2017, in a patient indicated for cardiac resynchronization therapy, in whom the implantation of a coronary sinus lead was not possible, and His-bundle pacing did not correct left bundle branch block [44]. In the original description, the pacing lead tip was advanced through the septum toward the left ventricle with successful identification of the left bundle branch which resulted in the normalization of interventricular conduction delay, low and stable pacing thresholds, with improvements in LVEF and symptoms. With left bundle branch area pacing, the right ventricular lead is delivered distal to the His bundle into the interventricular septum with the helix rotated deep into the myocardium to achieve cathodal (subendocardial) stimulation of the left ventricle. It is yet unclear whether specific left bundle pacing, left bundle branch area (left bundle branch and myocardium), or left ventricular septal pacing is sufficient to optimize coordinated activation of the left ventricular myocardium. True left bundle branch pacing leads to delayed activation of the right ventricle, such that interventricular delay can be worsened, whereas left bundle branch area pacing might provide an optimal balance between inter- and intraventricular timing and has become the accepted approach in most centers. A recent European consensus statement promotes the transventricular septal technique as the ideal method of achieving LBBAP, with the distal His-bundle potential used as an anatomical marker and observation of transition of the paced QRS morphology to right bundle branch pattern conduction delay and presence of left bundle branch potential during lead advancement into the interventricular septum as electrical markers of successful left bundle branch pacing [45].

Early comparisons with right ventricular pacing have been favorable. LBBAP results in shorter QRS durations compared to apical or septal positions, low pacing thresholds and stable lead impedances, comparable to right ventricular pacing [46,47]. China was the first country to enthusiastically adopt LBBAP in clinical practice, maintaining a centralized registry which already included ~5,000 conduction system pacing implants in 2018, ~80% of which were LBBAP [48]. Adoption in other countries has been more cautious, with early reports from the United States from around this time describing their initial experience in 100 patients with either left bundle branch block, right bundle branch block or nonspecific interventricular conduction delay, who had either indications for pacing or cardiac resynchronization therapy with failure to implant a coronary sinus lead [49]. In this cohort, implantation of a left bundle branch area lead was successful in 93% of patients, with consistently low pacing thresholds, although there were three early lead displacements and three instances of left ventricular septal perforation requiring lead repositioning in an adjacent position.

Given the current extended indication for cardiac resynchronization therapy in patients with LVEF 36–50% in Europe and the United States, it is appropriate to consider LBBAP for bradycardia in this group compared with both right ventricular and biventricular pacing. In patients with HFrEF and left bundle branch block, the extent of QRS narrowing is related to the degree of improvement in left ventricular function. Furthermore, a narrow QRS even in the presence of marked left ventricular dysfunction and mechanical dyssynchrony is better than a paced beat delivered by cardiac resynchronization therapy [50–52]. It does seem logical, therefore, that in a patient requiring heart rate support, while biventricular stimulation is likely to worsen left ventricular dyssynchrony less than right ventricular pacing, the QRS duration achieved with biventricular pacing is not normal, and a narrower QRS utilizing the intrinsic conduction system might be associated with even less mechanical dyssynchrony. LBBAP does seem to provide a more physiological left ventricular electrical and mechanical stimulation [53]. Therefore, although the guideline indication for biventricular pacing for bradycardia with LVEF 36–50% is not universal, in these patients it is reasonable to compare outcomes of LBBAP with both biventricular and right ventricular pacing. On the other hand, in patients without left ventricular dysfunction, for whom the evidence and guidelines do not support biventricular pacing, trials are required to determine whether the benefit on clinical outcomes outweighs the additional procedure times and complications.

While randomized trials are awaited, more recent data from registry studies, including the Multicentre European Left Bundle Branch Area Pacing Outcomes Study (MELOS), reported outcomes for 2533 patients undergoing implantation of LBBAP in 14 European centers, including 1218 patients with atrioventricular block [54]. The overall success rate was 92.5% (comparable with coronary sinus lead implantation [55]). Of note, the reported 8.3% rate of complications related to the transseptal route of the LBBAP lead far exceeds the rate of heart failure hospitalization associated with right ventricular apical pacing, which itself is associated with a low risk of lead-related complications. On the other hand, serious concerns such as stroke due to left ventricular endocardial penetration seem not to be a major problem, with a rate of late septal perforation <0.5%.

### 3.3. Currently enrolling clinical trials evaluating conducting system pacing for bradycardia

While observational studies have confirmed the feasibility of His-bundle and LBBAP [49], given the lack of clarity with regard to clinical endpoints, current clinical guidelines are divided, with those from the United States providing a cautious recommendation for those who will require substantial ventricular pacing (20–40%) [56], while European guidelines make no recommendation. Clinical trials which aim to provide a definitive answer to whether and for whom LBBAP is preferable to right ventricular apical pacing are underway. PROTECT-HF (Physiological Vs Right ventricular pacing Outcome Trial Evaluated for bradycardia Treatment) is the largest of such studies, with a recruitment target of 2600 participants who have LVEF > 35% and an indication for pacing, aiming to compare conventional right ventricular apical pacing with His-bundle or LBBAP. Although this trial should be adequately powered to offer a head-to-head comparison with right ventricular apical pacing and has the potential to change practice, it is questionable whether the standard care arm should mandate right ventricular apical pacing for those with LVEF 36–49%, in whom up front cardiac resynchronization therapy is likely to represent standard of care in many centers [34]. PROTECT-UP (Physiological versus Right ventricular pacing outcome trial for bradycardia treatment upgrades) takes a different approach, and aims to randomize 155 participants previously implanted with standard pacemakers and approaching generator replacement who have pacemaker-associated heart failure, to a physiological pacing upgrade (conduction system pacing or biventricular pacing), or a like-for-like generator replacement.

A number of other studies comparing LBBAP with right ventricular pacing in patients with atrioventricular block and a high anticipated right ventricular pacing burden are underway, of which two are focussing on patients without left ventricular dysfunction (LEFT; *n* = 100, LEAP-Block; *n* = 458), while others include all patients with LVEF >35% or 40% (PHYSPAVB; *n* = 200, LEAD; *n* = 470, PROTECT-SYNC; *n* = 450, and OptimPacing; *n* = 683). LBBAP is also under investigation for other indications, including in the PACE-FIB (Heart Rate Regularization in Atrial Fibrillation and Heart Failure) study, which will randomize patients with heart failure with preserved or mildly reduced ejection fraction and permanent atrial fibrillation to either LBBAP followed by atrioventricular node ablation or a rate control strategy [57].

## 4. Devices for heart failure

### 4.1. Cardiac resynchronization therapy

The last three decades have seen significant advances in the medical management of HFrEF, with contemporary pharmacological therapy consisting of four classes of medications which improve life expectancy and reduce hospitalization rates [58]. However, the presence of conduction tissue disease, especially in the form of left bundle branch block, drives the progression of the syndrome [59] and is associated with less reverse remodeling [1]. Biventricular pacing, in which a lead is implanted into the right ventricular apex and another into a coronary sinus branch, is now accepted as standard therapy for patients with LVEF < 35% and left bundle branch block [34]. Clinical trials assessing cardiac resynchronization therapy across the range of symptoms and disease severity have shown consistent benefits in terms of symptoms and survival [6,55]. In fact, the major barrier to achieving more optimal outcomes is the underutilization of this proven technology, with implantation even in Europe only achieved in a third of eligible patients because of a combination of factors including reimbursement, physician inertia and patients characteristics [60]. One additional driver may be the commonly held belief that failure to achieve symptomatic improvement or reverse remodeling equates to treatment failure [61,62]. The counterarguments to this is that in the setting of a progressive disease such as HFrEF, the achievement of stability is a positive outcome which can only be assessed in comparison to the unimplanted [63], and also that there may be a ceiling effect dependent upon the stage of the condition at which biventricular pacing is offered [64]. Nevertheless, there are clearly a subgroup of patients who derive little objective improvement following biventricular pacing, and it is an outstanding question whether ‘more optimal’ resynchronization (QRS narrowing) could improve outcomes further. Hence, currently proposed indications for conduction system pacing range from the approach being an alternative to conventional biventricular pacing for those in whom a conventional CS lead could not be implanted, or does not result in clinical improvement, to the approach representing a new standard of care.

### 4.2. His-bundle pacing as an alternative strategy to cardiac resynchronization therapy

The potential of His-bundle pacing as a non-conventional method of delivering cardiac resynchronization therapy is appealing, given the potential to produce more physiological electrical activation of the intrinsic conduction system. Observational data have suggested His-bundle pacing may have merit, both for those where the implantation of a coronary sinus lead is not possible or as an alternative to biventricular pacing, due to observed with reductions in QRS duration, improvements in LVEF and symptoms [65].

Three clinical trials have compared His-bundle pacing to standard biventricular pacing, assessing hemodynamic endpoints with limited patient follow-up. In a small pilot, cross-over study, 29 patients eligible for cardiac resynchronization therapy received a right atrial lead, defibrillator lead, as well as a coronary sinus lead, and His-bundle lead connected via a Y-adaptor to the left ventricular port of the generator. Participants were then randomized to a 6-month cross-over comparing biventricular pacing to His-bundle pacing. Only 12 patients completed the cross-over analysis at 1-year, and there were similar improvements in symptoms, 6-min walk distance, and LVEF with both approaches [66]. Two later pilot studies, His-SYNC (His-Bundle Pacing versus Coronary Sinus Pacing for Cardiac Resynchronization Therapy) [67] and His-Alternative (A Randomized Trial of His Pacing Versus Biventricular Pacing in Symptomatic HF Patients with Left bundle Branch Block) [68], enrolled patients indicated for cardiac resynchronization therapy according to current criteria. In these trials, His-bundle pacing was associated with comparable reductions in QRS duration, and there were no differences in hemodynamic or clinical endpoints, although pacing thresholds were significantly higher in the His-bundle group. A limitation of this trial was the high crossover rate, with 48% of patients randomized to His-bundle pacing ultimately requiring conventional cardiac resynchronization therapy and 26% of those randomized to biventricular pacing subsequently receiving His-bundle pacing. The most commonly reported reason for the crossover was inadequate shortening of the RS duration. ALTERNATIVE-AF (Comparison of His-bundle pacing and bi-ventricular pacing in heart failure patients with atrial fibrillation who need atrioventricular node ablation) is the only study to date to have demonstrated modest differences in LVEF (+3.5% vs −2.4%;*p* = 0.015) with the receipt of His-bundle pacing. This crossover trial compared His-bundle pacing with biventricular pacing for patients undergoing atrioventricular node ablation for atrial fibrillation, in whom the implantation of biventricular pacing is an accepted alternative to right ventricular pacing [69].

### 4.3. Left bundle branch area pacing as an alternative to cardiac resynchronization therapy

Observational studies have confirmed the feasibility of providing LBBAP as a novel strategy to provide a more physiological method of pacing. In these studies, LBBAP seems to overcome the hurdles of by His-bundle pacing, with low and stable pacing thresholds and high success rates even in patients with interventricular conduction delay. This has sparked interest as to whether LBBAP could be feasibly used as a method of delivering non-conventional cardiac resynchronization therapy. As an emerging technique, there is little robust data to support this indication, with much of the available evidence coming from small and highly selected cohorts in non-randomized observational studies. These have suggested that LBBAP improves QRS duration, LVEF, and symptoms, both where LBBAP is used as an initial strategy [70] or where biventricular pacing has been unsuccessful [71]. These studies do not offer a direct comparison with biventricular pacing, and implant practice varied with some patients receiving LBBAP in addition to coronary sinus lead placement. More recently, a large, observational case-control study, including 1778 patients from 15 international centers compared clinical outcomes between those receiving biventricular pacing and LBBAP in patients with Class I or II indications for cardiac resynchronization therapy [72]. In this study, patients may have received conventional biventricular pacing, LBBAP, or LBBAP-optimized cardiac resynchronization therapy, in which a coronary sinus lead could also be implanted at the operator’s discretion. The receipt of LBBAP was associated with a lower risk of death or heart failure hospitalization. However, by only including implanted patients, this study could not comment on procedure success rates nor account for unmeasured confounders. Of note, the higher use of implantable cardioverter defibrillators in those receiving conventional biventricular pacing suggests these two cohorts may have distinct risks of adverse events.

LBBAP has the potential to correct conduction delay caused by left bundle branch block; however, a potential limitation is the inability to correct other forms of interventricular conduction delay. Left bundle branch-optimized CRT (LOT-CRT) has been proposed as an alternative strategy to LBBAP, for both those with nonspecific interventricular conduction delay, as well as those with suboptimal electrical response to conventional biventricular pacing. In LOT-CRT, a conventional coronary sinus lead is placed alongside a lead implanted into the left bundle branch area. Small observational studies have suggested that in patients with nonspecific conduction delay, as well as those with suboptimal response to biventricular pacing (who often had nonspecific conduction delay or right bundle branch block), electrical and echocardiographic were more favorable with LOT-CRT [73,74].

The limitations of observational studies in a chronic incurable condition such as heart failure mean that to fully understand if LBBAP or LOT-CRT is comparable (or even superior) to conventional cardiac resynchronization therapy, randomized data are needed. To date, LBBAP-RESYNC (Left Bundle Branch Pacing Versus Biventricular Pacing for Cardiac Resynchronization Therapy) is the only randomized trial assessing LBBAP as an alternative to biventricular pacing [75]. The study enrolled 40 patients with left bundle branch block and non-ischemic cardiomyopathy. In those randomized to LBBAP, either a coronary sinus pacing lead or a right ventricular defibrillator lead was also implanted as a backup. There were greater improvements in LVEF (mean difference 5.6%; *p* = 0.039) in those randomized to LBBAP and lower natriuretic peptides, although counter-intuitively QRS durations were similar. There were high rates of crossover (10% in those randomized to LBBAP and 20% in those randomized to biventricular pacing) and patient-orientated outcomes were not different. It is unlikely that clinical practice guidelines will embrace LBBAP for this indication in the absence of further trials powered by clinical outcomes.

### 4.4. Implantable cardioverter defibrillators

Sudden death is a major but somewhat preventable contributor to mortality in HFrEF [76], with those who have ischemic heart disease, severely impaired left ventricular function, or prior history of ventricular tachyarrhythmia being at the greatest risk [77]. As such, patients with HFrEF currently indicated for cardiac resynchronization therapy commonly receive an implantable cardioverter defibrillator (ICD) to reduce the risk of sudden death. Although landmark trials of ICDs were conducted in an era with less optimal medical therapy [78,79] and included mostly patients with an ischemic etiology, and in the face of information from DANISH (Danish Study to Assess the Efficacy of ICDs in Patients with Non-ischemic Systolic Heart Failure on Mortality) where ICDs reduced sudden but not all-cause mortality, these devices continue to be recommended as primary prevention for all patients expected to live at least 1-year [4].

The decision to offer a defibrillator involves an individualized evaluation of the balance of risk between sudden cardiac and non-cardiovascular death. Factors such as arrhythmic syncope, myocardial scar, and sustained ventricular tachycardia increase the risk of sudden death, while non-cardiovascular co-morbidities or frailty increase the competing risk of death from another cause. Although there are specific disadvantages to offering a defibrillator, globally around 80% of cardiac resynchronization therapy devices implanted for HFrEF continue to have a defibrillator component [80]. As yet, there is no simple and commercially available method of providing an ICD with conduction system pacing. Left bundle branch-optimized ICD (LOT-ICD) is a novel approach proposed to address this shortcoming, in which cardiac resynchronization therapy is achieved using LBBAP, with an additional right ventricular apical ICD lead. The lack of setting specific generators is overcome by using a DF-1/IS-1 ICD lead attached to a dual chamber IS-1/DF-1 generator. Currently, experience of this technique is limited to case reports and small case series, and the requirement for an additional right ventricular lead may be impractical given the high cross-over rate to conventional CRT due to the difficulty in delivering LBBAP to patients with an indication for an ICD who are likely to have a high burden of myocardial scar [81].

## 5. How should conduction system pacing fit into the heart failure pathway?

**Figure 1.**
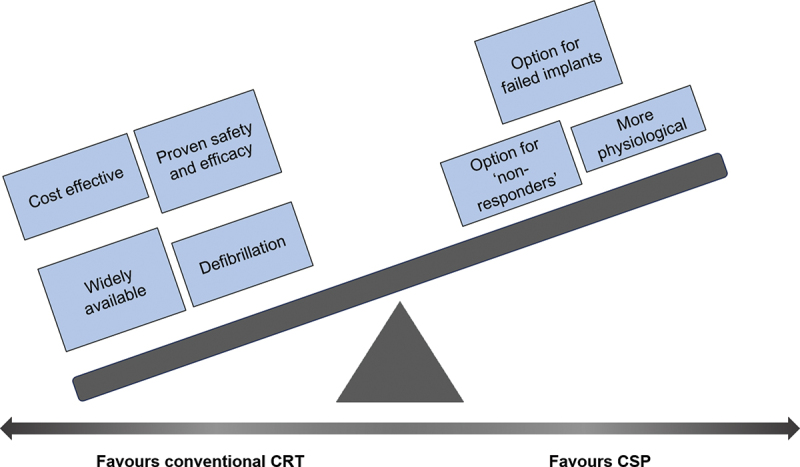
The current evidence is still overwhelmingly in favor of conventional CRT with BiV pacing in heart failure. Until more robust evidence with long term safety data is available, CSP should only be considered as an alternative to conventional CRT in select cases (CSP-CRT conduction system pacing as a form of CRT).

The totality of the available evidence supporting conduction system pacing as an alternative strategy for treating conduction delay in heart failure pales in comparison to the robust data supporting conventional biventricular pacing (Figure 1). Current guidelines limit its role to a bailout option where the implantation of a coronary sinus lead is not possible. Conventional cardiac resynchronization therapy is therefore likely to remain the standard of care for the foreseeable future [34]. Specific concerns regarding conduction system pacing include the lower success rates of His-bundle pacing where there is interventricular conduction delay, higher pacing thresholds, and for both modalities, the lack of available devices which are able to provide defibrillation without the need for additional leads [37,67]. The longevity of modern left ventricular leads is high, while extraction, including those with an active fixation mechanism, is straightforward, although there are specific complications such as coronary sinus dissection. On the other hand, there are legitimate concerns about the long-term safety of conduction system techniques, especially the unknown long-term consequences of deep septal leads [82] and also particularly their suitability for extraction.

**Figure 2.**
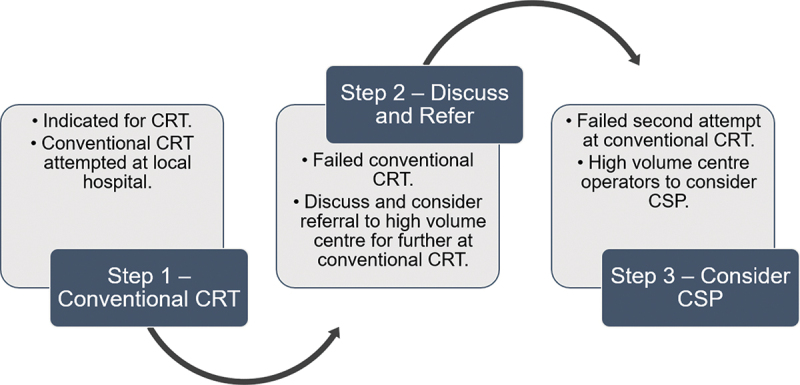
The current guidelines recommend the following series of steps when considering mode of delivery of CRT. The authors would add in that in the event of failure of initial attempt at CRT, there is a duty to refer patients to high volume centers with experienced operators to reattempt conventional CRT delivery before considering other options. To purely offer CSP from the outset would be doing our patients a disservice as the technique is still in its infancy and the long-term implications are poorly studied and understood.

Biventricular pacing is associated with high implant success rates and low risk of complications. Despite this, there is a subgroup of patients currently classified as ‘non-responders’ in whom conduction system pacing may be an alternative. There is also an inverse relationship between the individual operator volume and the rate of complications and procedure times, with implants performed by lower volume operators having 47% and 147% higher odds of developing mechanical complications or infections, respectively [83]. There is no reason to believe that conduction system pacing would be any different in terms of both response and complication rates, with experience particularly relevant for complex devices for which the potential to reduce implant failure is also a relevant consideration. Therefore, when conduction system pacing is used as an alternative to (failed) cardiac resynchronization therapy, these devices should be implanted at high-volume centers (Figure 2).

## 6. Conclusions

Conduction system pacing, including His-bundle pacing and LBBAP, is promising alternatives to right ventricular pacing, and may have a role in the heart failure pathway as alternatives to cardiac resynchronization therapy. However, until we have evidence from adequately powered clinical trials demonstrating its clear superiority to right ventricular pacing or non-inferiority to cardiac resynchronization therapy, these devices should not be standard of care. Currently, conduction system pacing should only be considered as an alternative to conventional cardiac resynchronization therapy where there is a failure to implant a coronary sinus leads by a high-volume operator, to ensure all patients have equitable access to evidence-based therapies.

## 7. Expert opinion

Conduction system pacing, including His-bundle pacing and left bundle branch area pacing (LBBAP) have the potential to be a step change in our approach to pacemaker therapy. These strategies avoid the disadvantages of standard right ventricular apical pacing for bradycardia and may offer a more physiological alternative to biventricular pacing for those with heart failure currently indicated for cardiac resynchronization therapy.

However, we have been here before. Enthusiasm for pacing the ventricle with short atrio-ventricular delays in dilated cardiomyopathy quickly turned to dismay; right ventricular septal pacing remains popular but clinical trials show us it is no better than standard apical pacing; cardiac resynchronization therapy is not an option for mechanical dyssynchrony in the absence of a broad QRS; and upfront biventricular pacing for bradycardia in unselected patients is of marginal benefit. Moreover, the rate of clinical heart failure in people with right ventricular pacing leads who develop left ventricular dysfunction is low and can be reduced by the provision of optimal medical therapy. It is also worth reflecting on the fact that each advance in pacemaker technique and technology has come with its own specific limitations and risk of complications, and conduction system pacing is unlikely to be any different.

It is not yet known whether conduction system pacing for bradycardia will influence patient-orientated outcomes compared to standard of care, whether it is truly better, easier, and safer than a coronary sinus lead and whether health-care systems can adapt to make it routine. It is likely, therefore, that standard approaches will remain widespread for many years, offering the opportunity to continue to collect outcome data in adequately powered randomized controlled trials.

We propose that, based upon current evidence, while the future for conduction system pacing is bright, whether it is truly a panacea which will solve all the problems of standard approaches, without heralding unique limitations, requires further study. The next decade will see improved success rates, the refinement of tools and techniques, and the urgently needed efficacy and safety data. Careful data collection in observational registries and controlled trials will clarify whether health-care systems and implant teams should adapt to offer conduction system pacing routinely for the treatment of bradycardia and heart failure, and whether it should be provided to selected patients or across the board.

Whichever direction the embedding of conduction system pacing into routine care takes, device therapy will remain a field in which personalization of both hardware and device programming, as well as operator experience, will remain key issues. Its more widespread adoption will require a foundation of solid evidence to ensure doctors, and patients can make informed decisions regarding the risks and benefits of the expanding landscape of options.
